# Occasional Non-Significance of Albuminuria

**Published:** 1878-08

**Authors:** 


### The Occasional Non-Significance of Albuminuria.

A writer in the *Lancet* observes that the presence of albumen in the urine, even in very minute quantities, is usually taken as conclusive evidence of such a condition of systemic derangement as deserves to be regarded as disease. Professor Leube of Erlangen, considering the fact that a trace of albumen is occasionally present in the urine of patients who present no other evidence of renal disease, examined the urine of a large number of men presumably in good health,soldiers on regular duty,in order to ascertain whether albumen could ever be detected in the urine of those who presented no other morbid symptoms. He examined the morning urine of 119 soldiers, and found a trace of albumen in that of five, in one a distinct quantity, in the others merely a faint, just recognizable, trace. In each case the mid-day urine also contained albumen, and in three the amount was larger than in the morning urine. So far the results are not surprising, since they would seem explicable on the theory that these soldiers were suffering from incipient renal disease, which, as is well known, may be unattended by any indication of deranged health. But it was found that albumen was present in fourteen soldiers out of 119 whose morning urine had been free from albumen. In all these instances the soldiers had undertaken arduous duty, marches or battalion drill, during the forenoon, but no food or drink had been taken during the exercise. No casts or blood were found in the deposit from the urine. The albumen soon disappeared with rest. This fact seems to show that in apparently healthy men exertion may induce slight albuminuria. This opinion was supported by an instance in which the urine of a soldier was for two days free from even a trace of albumen, but on the third, after a fatiguing march, showed obvious quantities of it.—*Med. and Surg. Reporter.*

